# Inhibition Mechanism of Mulberry Prenylated Flavonoids Sanggenone D/Kuwanon G Against α-Glucosidase and the Regulation of Glucose via GLUT4 Pathway

**DOI:** 10.3390/nu17091539

**Published:** 2025-04-30

**Authors:** Erwen Wu, Yanqing Zhu, Qingyi Wei, Huijie Lu, Yuxiao Zou, Fan Liu, Qian Li

**Affiliations:** 1 Key Laboratory of Functional Foods, Ministry of Agriculture and Rural Affairs, Guangdong Key Laboratory of Agricultural Products Processing; Sericultural & Agri-Food Research Institute, Guangdong Academy of Agricultural Sciences, Guangzhou 510610, China; 13070242926@163.com (E.W.); zhuyanqing@stu.scau.edu.cn (Y.Z.); zouyuxiao@gdaas.cn (Y.Z.); liufan1234@126.com (F.L.); 2 School of Food Science and Engineering, South China University of Technology, Guangzhou 510640, China; feweiqingyi@scut.edu.cn; 3 Institute of Animal Science, Guangdong Academy of Agricultural Sciences, Guangzhou 510640, China; luhuijie@mail2.sysu.edu.cn

**Keywords:** α-glucosidase, inhibition mechanisms, molecular docking, Western blotting, structure–activity relationship

## Abstract

Background: Inhibition of α-glucosidase activity is recognized as an effective strategy for managing type 2 diabetes. Methods: The inhibitory mechanisms of two kinds of mulberry flavonoids, namely sanggenone D and kuwanon G, on α-glucosidase were investigated and the hypoglycemic pathways were explored in the current study. Results: The outcomes indicate that sanggenone D (IC_50_: 4.51 × 10^−5^ mol/L) and kuwanon G (IC_50_: 3.83 × 10^−5^ mol/L) inhibited α-glucosidase activity by non-competition/anti-competition mixed inhibition and competitive inhibition, respectively. Moreover, the secondary structure of α-glucosidase was altered by static quenching and exhibited a decrease in α-helix and β-antiparallel content, and an increase in β-sheet content. Furthermore, the interaction forces between sanggenone D/kuwanon G and α-glucosidase were hydrophobic interactions and hydrogen bonds, as evidenced by molecular docking. The binding affinity, stability, and binding energy aligned with the results of IC_50_. Notably, the cyclization in sanggenone D structure resulted in a decrease in the number of phenolic hydroxyl groups and thus a reduction in the formation of hydrogen bonds, which ultimately diminished the binding affinity of sanggenone D to α-glucosidase. In addition, Western blot analysis further indicated that sanggenone D and kuwanon G regulated glucose metabolism by activating the GLUT4 pathway. Conclusions: The results provided useful reference for the application of sanggenone D and kuwanon G in hypoglycemic functional components.

## 1. Introduction

Diabetes mellitus is a metabolic condition resulting from impaired insulin secretion, characterized by elevated fasting blood glucose, excessive thirst, hunger, urination, and weight loss. Management of the condition generally involves ongoing medication to regulate blood sugar levels [1]. According to global statistics, approximately 9% of adults suffer from diabetes, with more than 90% of cases classified as type 2 diabetes mellitus (T2DM) [2]. The clinical manifestations of T2DM are hyperglycemia and insulin resistance. When the feedback pathway of insulin action and insulin secretion is destroyed, all insulin-sensitive organs in the human body will be greatly affected, leading to the failure of synthesize glycogen with glucose normally, and resulting in increased glucose concentration in the blood and the formation of hyperglycemia [3].

Alpha-glucosidase is a key digestive enzyme that degrades starch into monosaccharides, and many compounds show their biological activities of decreasing blood glucose by inhibiting α-glucosidase [4,5]. Currently, several α-glucosidase inhibitors, including acarbose, sitagliptin, and miglitol, are widely prescribed to manage diabetes [6]. However, these synthetic inhibitors are often associated with adverse effects such as hypoglycemia, gastrointestinal discomfort, increased insulin resistance, and hepatotoxicity. As a result, the search for safe, effective, and economical dietary anti-diabetic compounds is of great importance [7].

Mulberry is a valuable plant, and its different parts (leaves, branches, roots) have been widely utilized for centuries in the management of diabetes, rheumatics, arthritis, and other diseases [8,9]. Mulberry leaves contain polyphenolic compounds, such as flavonoids, which consist of two benzene rings connected by a heterocyclic six-membered pyrone ring. The structural properties of flavonoids play a crucial role in determining their biological functions, including antioxidant activity, cardiovascular protection, and anti-cancer potential [10,11]. Studies have shown that rats taking mulberry leaf extract for 5 weeks demonstrated reduced blood sugar levels in the body by 5%, and oral administration of 0.8 g and 1.2 g mulberry leaf powder significantly inhibited post-meal blood sugar and insulin secretion [12,13].

Mulberry resources are rich in prenylated flavonoids, such as sanggenone D and kuwanon G, each containing one prenyl group. Wen’s research indicated that compared to non-prenylated flavonoids, prenylated flavonoids exhibit significantly enhanced activities in areas such as immunomodulation, cardiovascular protection, and improvement in metabolic diseases. Moreover, the structure–activity relationship of a single prenyl group in flavonoids shows activity ranked as follows: *C-*8 prenyl > *C-*3 prenyl > *C-*6 prenyl [14]. Prenylation significantly enhanced the inhibitory effects of parent compounds like quercetin, genistein, and chalcone on α-glucosidase activity [15].

Despite these findings, research on the precise molecular target and inhibitory mechanism of sanggenone D and kuwanon G against α-glucosidase remains limited. In this study, enzymatic kinetics, Fourier transform infrared spectra, fluorescence spectroscopy, and molecular docking techniques were applied to explore the inhibitory effect and mechanism of α-glucosidase inhibition by sanggenone D and kuwanon G. Moreover, the high-glucose model of human hepatocellular carcinoma HepG2 cells was established to investigate the effect of sanggenone D and kuwanon G on reducing glucose level, and the pathway of their action was explored by Western blotting. The results would provide useful reference for the application of sanggenone D and kuwanon G in hypoglycemic functional food.

## 2. Materials and Methods

### 2.1. Materials

Alpha-glucosidase, acarbose, and p-nitrophenyl-α-D-glucopyranoside were sourced from Shanghai Yuanye Biotechnology Co., Ltd., Shanghai, China. Sanggenone D and kuwanon G were sourced from Baoji Chenguang Biotechnology Co., Ltd., Baoji, China, with a purity of >98%. HepG2 cells were sourced from Yuji Biotechnology Co., Ltd., Shanghai, China. Trypsin, fetal bovine serum, double antibody, non-essential amino acid, and high-glucose Dulbecco’s modified Eagle’s medium (DMEM) were sourced from Casbad, CA, USA. A CCK-8 kit, glucose assay kit, total cholesterol (TC) kit, and triglyceride (TG) kit were sourced from Nanjing Jiancheng Bioengineering Institute, Nanjing, China. Analytical-grade reagents with a purity of >99.8% were used for the experiment, along with ultra-pure water.

### 2.2. Inhibitory Activity

Samples of 50 μL serial dilution concentration (sangggenone D: 70.5, 141.1, 282.2, 564.3, 846.5, 1128.6 μM; kuwanon G: 72.18, 144.36, 288.72, 360.90, 433.08, 577.44 μM; acarbose: 38.72, 77.45, 116.17, 154.89, 193.62, 387.23 pM) ($Concentration\left(µg/mL\right)=Concentration(µM)\times MW(g/mol)\times 10^{-3}$) were put in a 96-well plate, then 50 μL of 1 U/mL α-glucosidase solution (prepared in pH 6.8, 0.1 mol/L PBS) was added, and the mixture was incubated at 37 °C for 10 min. Subsequently, 50 μL of 2 mmol/L p-NPG was added, and the mixture was incubated at 37 °C for 20 min. Finally, 50 μL of 1 mol/L Na_2_CO_3_ was added to an ice bath for 5 min to terminate the reaction. The absorbance was measured at 405 nm, and the α-glucosidase inhibitory activity was calculated according to Formula (1). All experiments were performed in triplicate.

(1) $$Inhibition\;rate\;of\;enzyme\;activity/\%=(1-\frac{A_{a}-A_{b}}{A_{c}-A_{d}})\times 100\%$$

*A_a_* is the absorbance of the sample group, *A_b_* is the absorbance of the sample background group (equal volume of buffer instead of enzyme solution), *A_c_* is the absorbance of the control group (equal volume of buffer instead of the sample solution), and *A_d_* is the absorbance of the control background group (equal volume of buffer instead of the sample and enzyme solution).

### 2.3. Inhibition Kinetics Assay

The inhibition assay of α-glucosidase was adapted from MORTEZA, with some modification [16]. A total of 50 μL of α-glucosidase solution (1 U/mL) was added to 96-well plates, and 50 μL of sample solution with different concentrations (sanggenone D: 0, 20, 100, 200 μg/mL; kuwanon G: 0, 10, 20, 40 μg/mL) was incubated at 37 °C for 10 min. Subsequently, different concentrations of pNPG solution were added and incubated at 37 °C for 20 min. Finally, sodium carbonate solution (50 μL, 2 mmol/L) was added to terminate the reaction. Colorimetry was performed at 405 nm using a spectrophotometer to determine the change in absorbance of the enzymatic reaction system. The reversible inhibitory effect types of sanggenone D and kuwanon G on the enzyme were analyzed by the Lineweaver–Burk equation. The relevant parameters were calculated using Formulas (2)–(4). Each experiment was conducted independently three times (*n* = 3).

(2) $$\frac{1}{V}=\frac{K_{m}}{V_{m}}\left(1+\frac{\left[I\right]}{K_{I}}\right)\times \frac{1}{S}+\frac{1}{V_{m}}(1+\frac{\left[I\right]}{K_{IS}})$$

(3) $$Slope=\frac{K_{m}}{V_{m}}+\frac{K_{m}[I]}{V_{m}K_{I}}$$

(4) $$Y-intercept=\frac{1}{V_{m}}+\frac{\left[I\right]}{K_{IS}V_{m}}$$

In the formula, *V* represents the reaction rate, and *V_m_* represents the maximum reaction rate. [*I*] is the concentration of the compound sample, and [*S*] is the substrate concentration. Km represents the Michaelis constant, and *K_I_* and *K_IS_* represent the inhibitory constants of the compound sample on α-glucosidase and the α-glucosidase–substrate complex, respectively.

### 2.4. Multi-Fluorescence Spectroscopy

A total of 100 μL of 1 U/mL α-glucosidase extract was mixed with PNPG solutions of different concentrations (sanggenone D: 0, 1, 5, 10, 20, 50, 60 μg/mL; kuwanon G: 0, 1, 5, 10, 20, 40, 60 μg/mL) and incubated at three temperatures of 298 K, 304 K, and 310 K for 5 min. Then, the mixture were scanned with fluorescence spectroscopy (SpectraMax i3x, Molecular Devices, LLC., San Jose, CA, USA). The excitation wavelength was set at 280 nm, the emission wavelength was 300–500 nm, and the widths of both excitation and emission slits were 5 nm. The fluorescence quenching parameters were calculated according to the following equations.

Stern–Volmer dynamic collision quenching equation:

(5) $$\frac{F_{0}}{F}=1+K_{SV}\left[Q\right]=1+K_{q}\tau_{0}\left[Q\right]$$

(6) $$lg\frac{F_{0}-F}{F}=lgK_{a}+nlg[Q]$$

F_0_ and F represent the fluorescence intensity peaks of α-glucosidase without and with the compound, respectively; [Q] is the sample concentration; and τ_0_ is the protein’s average lifetime (10^−8^ s).

### 2.5. FT-IR Measurements

Fourier transform infrared spectroscopy (VERTEX 7.0, BRUKER Co., Ltd., Bremen, Germany) was used to detect the secondary conformation of α-glucosidase before and after sample treatment by infrared amide I-type (1600 cm^−1^–1700 cm^−1^) curve fitting. In the experiment, the α-glucosidase (1 U/mL) was mixed with G (100 μg/mL) and D (150 μg/mL) in equal volumes [17].

### 2.6. Molecular Docking

Molecular docking techniques were employed to investigate the binding conformation of small molecules and proteins, and the binding energy, binding site, and binding mode of the interaction between sanggenone D, kuwanon G, and α-glucosidase were analyzed. The crystal structure of the α-glucosidase protein used for docking was acquired from the PDB database (PDB ID: 5NN4), while the 3D structures of the small molecules were retrieved from PubChem and subjected to energy minimization using the MMFF94 force field.

AutoDock Vina 1.1.2 software was used to perform molecular docking. Before docking, the α-glucosidase protein (EC 3.2.1.20) was processed using PyMol 2.5.2, which involved eliminating water molecules, salt ions, and small molecules. Subsequently, the docking box was positioned with its center aligned to the centroid of the ligand in the original crystal structure. Additionally, all processed small molecules and the receptor protein were converted into PDBQT format. During docking, the global search precision was set to 32, with all other parameters set to their default values. The docking conformation with the highest score was considered the binding conformation, and the docking results were subsequently visualized and analyzed using PyMol 2.5.2 [18,19].

The small molecule–protein complex obtained through docking was used as the initial structure for all-atom molecular dynamics simulations, which were carried out using AMBER 18 software [20]. Prior to the simulation, energy optimization of the system was performed using 2500 steps of steepest descent and 2500 steps of conjugate gradient methods. During the simulation, the nonbonded cutoff distance was set to 10 Å, and the particle mesh Ewald (PME) method was applied to calculate long-range electrostatic interactions. The SHAKE algorithm was used to constrain hydrogen bond lengths, and the Langevin algorithm was employed for temperature control. The collision frequency (γ), system pressure, and integration timestep were set to 2 ps^−1^, 1 atm, and 2 fs, respectively. Trajectories were saved every 10 ps for subsequent analysis.

The protein–ligand binding free energies for all systems were computed via the MM/GBSA approach [21]. MD trajectories of 90 to 100 ns were employed for the calculations in this research, with the detailed formula as follows.

(7) $$∆G_{bind}=∆G_{complex}-(∆G_{receptor}+∆G_{ligand})\;\;\;\;\;\;\;\;=∆E_{internal}+∆E_{VDW}+∆E_{elec}+∆G_{GB}+∆G_{SA}$$

In Formula (7), $∆E_{internal}$, $∆E_{VDW}$, and $∆E_{elec}\;$represent internal energy, van der Waals action, and electrostatic interaction, respectively. Internal energy includes *E_bond_*, *E_angle_*, and *E_torsion_*. $∆G_{GB}$ and $∆G_{SA}$ are collectively referred to as the free energies of solvation. *G_GB_* is the free energy of polar solvation, and *G_SA_* is the free energy of non-polar solvation.

### 2.7. Cell Experiment

#### 2.7.1. HepG2 Cell Cultivation

DMEM complete medium was configured with 88% DMEM high glucose medium, 10% fetal bovine serum, 1% double antibodies, and 1% non-essential amino acids. HepG2 cells were seeded in the complete medium and incubated at 37 °C with 5% CO_2_. When the cell confluence grew to 80–90%, the passage was carried out at a ratio of 1:3. According to the method of Cao et al., a hyperglycemic model was established through insulin induction [22].

#### 2.7.2. Toxicity Test of HepG2 Cells

HepG2 cells in the logarithmic growth stage were inoculated with 4 × 10^4^ cells per well in a 96-well plate and cultured at 37 °C and 5% carbon dioxide for 24 h. After the cell coverage reached 80%, 100 µL of sanggenone D and kuwanon G with different concentrations were added for 24 h. Then, 100 µL CCK-8/complete culture solution (1:9) was added to each well, and the absorbance at 450 nm was measured by an enzymoleter after 1 h of culture. The toxicity test was conducted in triplicate.

(8) $$Cell\;survival\;rate\;(\%)=\frac{A_{sample}}{A_{control}}\times 100\%$$

#### 2.7.3. Determination of Cellular Glucose, TC, and TG Content

The sample concentration (sanggenone D: 20, 80 μg/mL; kuwanon G: 20, 80 μg/mL) used in this experiment was determined by a CCK8 experiment. HepG2 cells were inoculated with 2 × 10^5^ in a 12-well plate and cultured for 24 h. After being attached to the well, the samples containing different concentrations were replaced and the cells were treated for 24 h. The relevant indexes were determined by the kit method according to the instructions of the manufacturer [23]. Each measurement was performed in triplicate.

#### 2.7.4. Western Blotting

The tissue proteins were extracted with cold total protein extraction buffer (TPEB) [98% RIPA buffer (Solarbio, Beijing, China), 1% phosphatase inhibitor mix (Sangon Biotech, Shanghai, China), and 1% protease inhibitor mix (Cell Signaling Technology, Danvers, MA, USA)], and protein concentration was determined by the bicinchoninic acid assay (BCA) method. A total of 20 μg of protein samples were separated by SDS-PAGE and transferred to a polyvinylidene difluoride (PVDF) membrane. The membrane was washed with PBST for 5 min, then incubated with 5% skim milk at room temperature for 1 h. Subsequently, the membrane was cleaned with PBST five times for 5 min and incubated overnight at 4 °C with the primary antibodies [ACC (Zenbio #381131; 1:750 dilution), AMPK (Zenbio #380431; 1:500 dilution), p-AMPK (Zenbio #340763; 1:750 dilution), CPT-1 (proteintech #15184-1-AP 1:3000 dilution), and GLUT4 (Zenbio #347063; 1:1000 dilution)]. Finally, the membrane was incubated with the secondary antibody (Abmart #T40104, 1:3000 dilution) at room temperature for 1 h. Chemiluminescent visualization of the protein bands was achieved using an ECL system (Millipore, Burlington, MA, USA) and documented with Image Lab software for PC Version 6.1 (Bio-Rad, South Granville, NSW, Australia). Densitometric analysis of the resulting bands was conducted using ImageJ 2 software.

### 2.8. Statistical Analysis

The experiments were conducted in triplicate, and data analysis was performed with GraphPad Prism 9.5 software (GraphPad Software Co., Ltd., Santiago, CA, USA). One-way ANOVA was used to assess differences, with statistical significance set at *p* < 0.05.

## 3. Results and Discussion

### 3.1. α-Glucosidase Inhibition Rate

**Figure 1:**
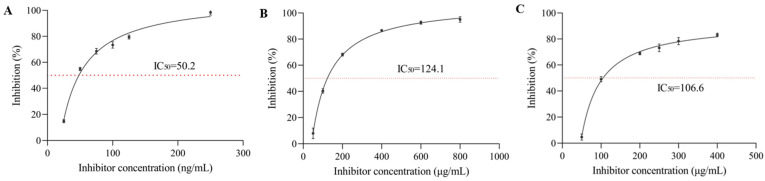
The inhibitory effect of different concentrations of (**A**) acarbose, (**B**) sanggenone D, and (**C**) kuwanon G on α-glucosidase activity.

The properties of essential groups in enzyme molecules are changed by the influence of certain substances, resulting in a reduction or loss of enzyme activity, called inhibition. As shown in Figure 1, the inhibition rate of α-glucosidase by acarbose (positive control), sanggenone D, and kuwanon G increased with higher compound concentration. The semi-inhibitory concentration values of acarbose, sanggenone D, and kuwanon G were 3.10 × 10^−7^ mol/L, 4.51 × 10^−5^ mol/L, and 3.83 × 10^−5^ mol/L, respectively, which indicated that kuwanon G had higher inhibitory activity on α-glucosidase than sanggenone D, but lower than that of acarbose. Although there is a certain gap compared to positive drugs, the activity is relatively strong when compared with other reported natural product compounds. For example, the IC_50_ of ferulic acid for α-glucosidase was 0.866 mg/mL [24], and the IC_50_ of the dodecyl-acylated derivatives of phlorizin and polydatin for α-glucosidase were 55.10 and 70.95 μM, respectively [25]. Compared with kuwanon G, sanggenone D was cyclized, which weakened the activity of the isopentenyl group and reduced one phenolic hydroxyl group, resulting in less hydrogen bonding with amino acid residues at the active site of α-glucosidase, herein, the decreased inhibitory activity, which is consistent with the findings of Sepehri et al. and was further validated in the molecular docking simulations in Section 3.5.2 [26].

### 3.2. Inhibition Type on α-Glucosidase Activity

**Figure 2:**
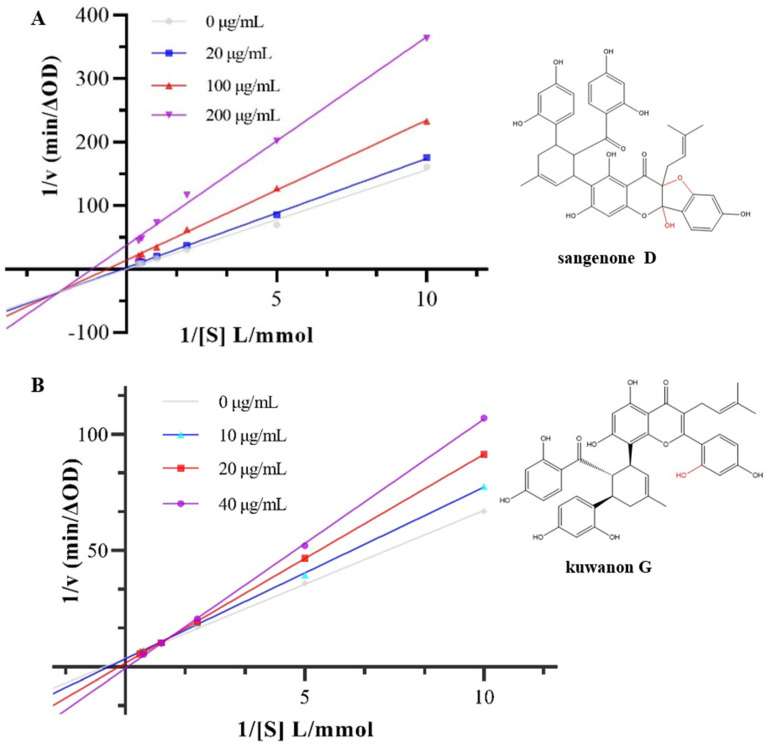
Inhibition kinetics of hyperoside on α-glucosidase by Lineweaver–Burk plots for (**A**) sanggenone D and (**B**) kuwanon G.

The inhibition type of α-glucosidase was determined according to the intersections in the Lineweaver–Burk diagram [27]. The Lineweaver–Burk curves of sanggenone D and kuwanon G with α-glucosidase are exhibited in Figure 2. The double reciprocal curves of different concentrations of sanggenone D intersected in the third phase quadrant. The slope of the line increased with the increase in sanggenone D concentration, while both K_M_ and the maximum reaction velocity (Vm) decreased, which indicated mixed inhibition of noncompetitive and anticompetitive [28]. The double reciprocal curves of kuwanon G intersected in the first quadrant of the phase diagram. With the increase in kuwanon G concentration, the K_M_ value increased, while the Vm value decreased, which represented the competitive inhibition type [17]. The curves in Figure 2 show a strong linear correlation, suggesting that the two phenolic compounds interacted with α-glucosidase through one binding site or a group of binding sites [29]. The reason for the different inhibition types might be that cyclization led to a change from a single competitive inhibition to a mixed inhibition, which weakened its inhibitory effect. However, due to little change in the overall structure, cyclization did not lead to a change in the number of inhibitory sites, which was still one binding site or a group of binding sites. According to Table 1, the Kis values of sanggenone D and kuwanon G were lower than the K_i_ values, suggesting that their interaction with the α-glucosidase–substrate complex was weaker than with the α-glucoglycinase–substrate complex [30].

### 3.3. Fluorescence Spectroscopy Analysis of Binding Mechanism and Properties

**Figure 3:**
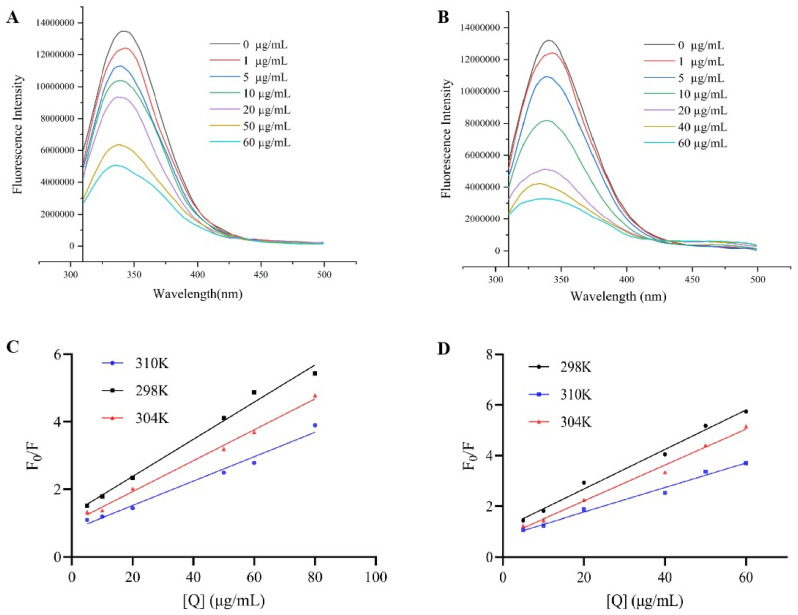
The effect of different concentration compounds on florescence spectra (**A**) sanggenone D and (**B**) kuwanon G; Stern–Volmer plots indicating α-glucosidase fluorescence quenching at different temperature for (**C**) sanggenone D and (**D**) kuwanon G.

The aromatic amino acids in α-glucosidase produced endogenous fluorescence when exposed to a specific excitation wavelength. The fluorescence intensity together with the position of the maximum emission wavelength were affected by the enzyme folding state and the environment surrounding the aromatic amino acid residues [31]. As shown in Figure 3A,B, under 280 nm excitation, the α-glucosidase solution displayed notable fluorescence between 300 and 500 nm, with the peak occurring at 340 nm. The fluorescence intensity of α-glucoglycase declined progressively as the concentration of sanggenone D and kuwanon G increased, and the maximum emission wavelength of the mixed solution shifted blue with regularity. These results indicate that sanggenone D and kuwanon G could quench the endogenous fluorescence of the enzyme by interacting with α-glucosidase.

Fluorescence quenching occurs in two forms: static quenching and dynamic quenching. In static quenching, non-fluorescent products are formed through the interaction of quenchers with fluorophores, with the quenching constant decreasing as temperature rises. Dynamic quenching occurs when excited fluorescent molecules collide with quenchers, leading to a decrease in fluorescence intensity, and its quenching constant increases with rising temperature [32]. The type of Stern–Volmer quenching curve, the change in static quenching constant at different temperatures, and the apparent quenching rate constant are an important basis for judging the quenching type [33]. The Stern–Volmer quenching curve exhibited a nice linear correlation when F0/F was plotted against [Q], and the quenching constant (Ksv) value decreased as the temperature increased. Moreover, the Ksv value aligned with the α-glucosidase inhibitory activity, following the order kuwanon G > sanggenone D (Figure 3C,D). In addition, the quenching rate constant (Kq) values for both compounds exceeded the maximum diffusion-controlled quenching constant of 2.0 × 10^10^ L/(mol·s), illustrating that the quenching of α-glucosidase by sanggenone D and kuwanon G were of the static quenching type [34]. As shown in Table 2, Ka value was negatively correlated with temperature, which is consistent with the change in Ksv, indicating that sanggenone D, kuwanon G, and α-glucosidase form an unstable complex. The binding force of the interaction between the two was destroyed by the increase in temperature, and the stability of the complex decreased with the increase in temperature, which is in accordance with the general characteristics of the static quenching type. In addition, at the experimental temperature, the value of n was close to 1, so it could be inferred that there was only one binding site on the α-glucosidase for sanggenone D and kuwanon G.

### 3.4. FT-IR Analysis

**Figure 4:**
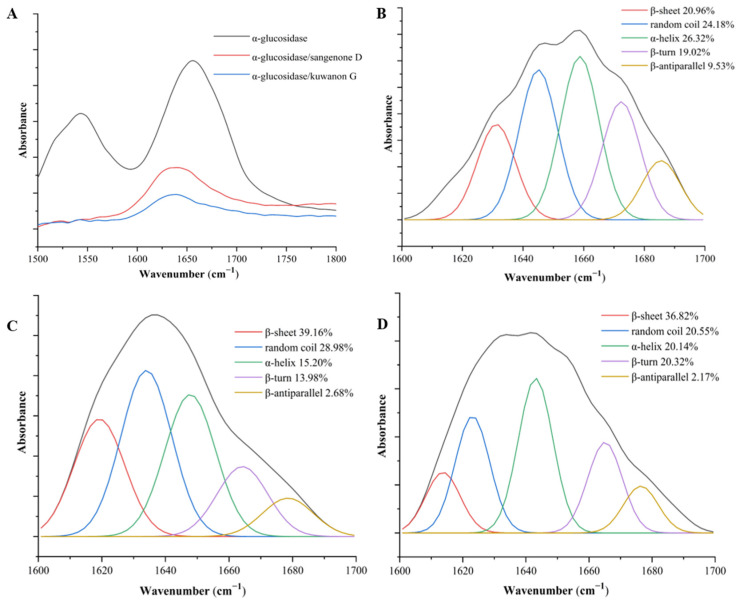
The FT-IR spectra of the α-glucosidase inhibitor. (**A**) The effect of sanggenone D and kuwanon G on the secondary conformation of α-glucosidase. (**B**) The α-glucosidase black control, (**C**) sanggenone D, (**D**) kuwanon G.

The interaction with phenolic compounds induced changes in the secondary structure of proteins [35]. The effects of sanggenone D and kuwanon G on the secondary conformation of α-glucosidase were analyzed using FT-IR spectroscopy. Because the amide Ι band produced by the C=O double bond stretching vibration is more responsive to changes in protein secondary structure than the amide ΙI band, this study mainly used the amide Ι band to quantitatively analyze the change in α-glucosidase secondary structure [36]. As shown in Figure 4A, after adding sanggenone D and kuwanon G to α-glucosidase, the peak position of the amide Ι band moved left from 1656 cm^−1^ to 1639 cm^−1^ and 1637 cm^−1^, respectively. Moreover, the free α-glucosidase contained 26.32% α-helix, 20.96% β-sheet, 19.02% β-turn, 9.53% β-antiparallel, and 24.18% random coil, respectively (Figure 4B). With the addition of sanggenone D and kuwanon G, the α-helix of the α-glucoglycase complex was reduced to 15.20% and 19.46%, β-antiparallel was reduced to 2.67% and 2.17%, and β-sheet was increased to 39.16% and 36.82%, respectively. These findings indicate that the secondary conformation of α-glucosidase was altered by sanggenone D and kuwanon G (Figure 4C,D). The secondary structure around the catalytic site of α-glucosidase was crucial for maintaining enzyme activity stability [37]. With the addition of inhibitors, α-helix content dropped significantly and β-sheet content rose obviously, which were speculated to relate to the decrease in α-glucosylase activity. The effect of sanggenone D and kuwanon G on secondary structure was obviously different, which might be the reason for their different inhibitory activities on α-glucosidase.

### 3.5. Molecular Docking Results

#### 3.5.1. Docking Mode Analysis

To further investigate the inhibition mechanism and exacted binding site on α-glucosidase, sanggenone D and kuwanon G were molecular-docked with α-glucosidase. The negative binding affinity indicated spontaneous binding between the compound and α-glucosidase, with a larger absolute value indicating a stronger binding force and an improved docking effect [38]. In this study, the values of the binding affinity of sanggenone D/α-glucosidase and kuwanon G/α-glucosidase were −7.73 and −7.94 kcal/mol, respectively, indicating that kuwanon G had a better inhibitory effect on α-glucosidase, which was consistent with the previous IC_50_ results.

**Figure 5:**
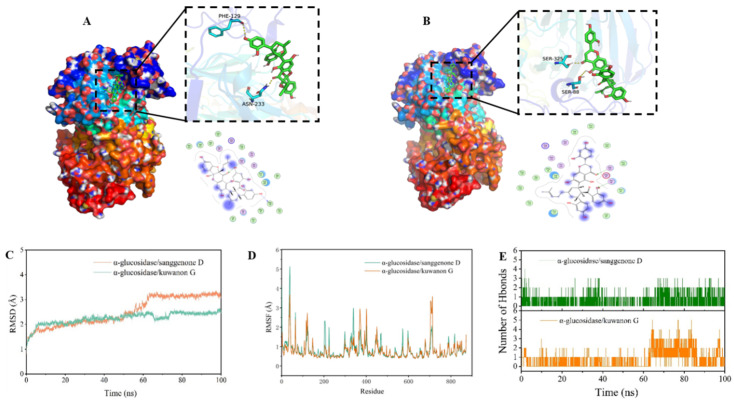
3D binding diagram and molecular dynamics simulation of α-glucosidase with sanggenone D and kuwanon G. (**A**) Sanggenone D, (**B**) kuwanon G, (**C**) root mean square deviation (RMSD), (**D**) root mean square fluctuation value (RMSF) based on molecular dynamics simulation, (**E**) number of hydrogen bonds.

As demonstrated in Figure 5, sanggenone D and kuwanon G attached to the active pocket inside the α-glucosidase protein. As can be observed from the mutual detail map, the binding sites of sanggenone D complex were PRO-253, VAL-230, ALA-327, PRO-326, PRO-131, VAL-236, PH-90, PRO-85, VAL-84, PH-129, ASN-233, SER-251, SER-88, and SER-325 amino acid construction. Among them, there was hydrophobic interaction with PRO-253, VAL-230, ALA-327, PRO-326, PRO-131, VAL-236, PH-90, PRO-85, VAL-84, and PH-129, and hydrogen bonding with PH-129 and ASN-233 (Figure 5A). In the same way, the binding sites of kuwanon G complex were VAL-230, PRO-253, LEU-252, PRO-326, PRO-85, VAL-84, VAL-236, PH-90, ALA-237, PRO-238, PRO-130, PRO-131, SER-325, and SER-88 amino acid construction. Among them, hydrophobic effects were observed between kuwanon G and VAL-230, PRO-253, LEU-252, PRO-326, PRO-85, VAL-84, VAL-236, PH-90, ALA-237, PRO-238, PRO-130, and PRO-131, and hydrogen bonding occurred with SER-325 and SER-88 at the site (Figure 5B). These interactions were the primary factor that ensures the stable combination between small molecules and α-glucosidase. In a word, these results indicated that the compounds interacted with the amino acid residues of α-glucosidase, thereby occupying the active site and inhibiting the activity of the enzyme. Moreover, the hydrophobic forces and hydrogen bonds were the main driving forces of the interaction [39].

#### 3.5.2. MD Simulation

The root mean square deviation (RMSD) in molecular dynamics simulations indicates the movement of the complex. A higher RMSD with larger fluctuations suggests more intense motion, while a lower RMSD indicate less movement [40]. As shown in Figure 5C, RMSD fluctuated less during the simulation of α-glucosidase/sanggenone D and α-glucosidase/kuwanon G complexes, which meant that the complex structure did not disintegrate, indicating a high stability in the binding of the small molecules to the protein. Additionally, the fluctuation in α-glucosidase/kuwanon G was relatively small, suggesting that kuwanon G bound more stably to the protein, with a better inhibition effect, which was consistent with the results of IC_50_.

In molecular dynamics simulations, the root mean square fluctuation (RMSF) indicated changes in protein flexibility [41]. After binding to the protein, the drug typically reduces the protein’s flexibility so as to stabilize the protein and play the role of inhibiting enzyme activity. As illustrated in Figure 5D, apart from the local region of the protein, the RMSF of the protein was less than 2 angstroms, indicating that the main structural rigidity of α-glucosidase was very high, which might have been due to the binding effect of the enzyme and the small molecules sanggenone D and kuwanon G with inhibitory effects.

Hydrogen bonding was among the strongest types of non-covalent interactions. The hydrogen bond count between ligand molecules and proteins was monitored over 100 ns during the molecular dynamics simulations. As depicted in Figure 5E, the number of hydrogen bonds formed by α-glucosidase/sanggenone D and α-glucosidase/kuwanon G in the simulation process ranged from 0 to 6. Most of the α-glucosidase/sanggenone D data were concentrated within 1–2, while most of the α-glucosidase/kuwanon G complex data were concentrated within 2–3. In general, the inhibitory activity of flavonoids on α-glucosidase was correlated with the quantity and location of hydroxyl functionalities in the compounds, which in turn affected the number of hydrogen bonds [34]. According to the present result, kuwanon G formed more hydrogen bonds with the α-glucosidase than sanggenone D, which contributed to understanding why kuwanon G had a better inhibitory effect than sanggenone D.

#### 3.5.3. MM–GBSA Result

The binding energy was computed using the MM–GBSA method based on molecular dynamics simulation trajectories, offering a more precise assessment of the interaction between small molecules and target proteins. As shown in Table 3, the combined energies of α-glucosidase/sanggenone D and α-glucosidase/kuwanon G were −9.83 ± 2.44 and −13.83 ± 2.94 kcal/mol. A negative number indicated that both molecules had an affinity for the target protein, and a lower value for α-glucosidase/kuwanon G complex represented a stronger binding than for α-glucosidase/sanggenone D. In addition, the main contribution of the α-glucosidase/sanggenone D and α-glucosidase/kuwanon G combinationswas van der Waals energy.

### 3.6. CCK-8 Assay

**Figure 6:**
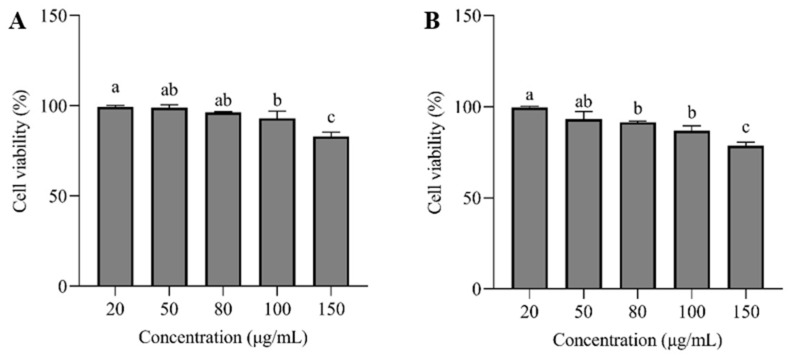
Survival rate of HepG2 cells treated with (**A**) sanggenone D and (**B**) kuwanon G. Note: Data are presented as mean ± standard deviation (SD) from n = 3 independent experiments. Different letters (a, b) indicate significant differences between groups (*p* < 0.05).

Cell survival assay is essential for assessing sample concentration and cytotoxicity. The cytotoxicity test results of HepG2 treated with sanggenone D and kuwanon G are shown in Figure 6. When the concentrations of sanggenone D and kuwanon G were less than 100 and 80 μg/mL, respectively, the survival percentage of HepG2 cells remained above 90%, reflecting that sanggenone D and kuwanon G were safe for HepG2 cells within this concentration range. This provided a reference for selecting the appropriate concentration range for subsequent experiments.

### 3.7. Glucose, TC, and TG Contents

**Figure 7:**
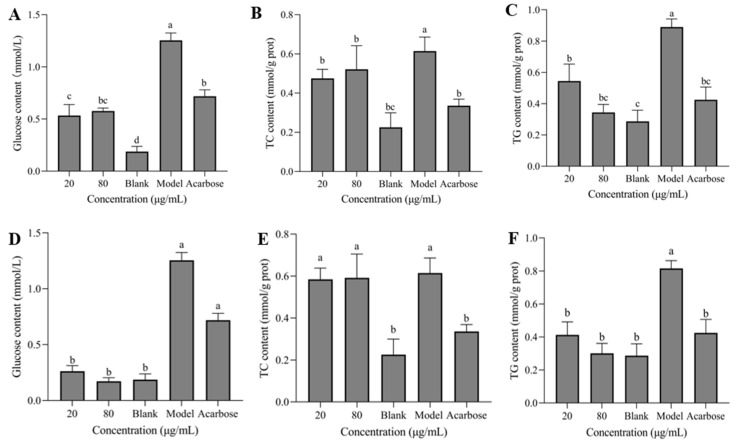
Effect of sanggenone D on glucose content in supernatant (**A**) and intracellular TC (**B**) and TG (**C**) content; effect of kuwanon G on glucose content in supernatant (**D**) and intracellular TC (**E**) and TG content (**F**). Note: Data are presented as mean ± standard deviation (SD) from n = 3 independent experiments. Different letters (a, b) indicate significant differences between groups (*p* < 0.05).

In Figure 7A–F, the model group showed significantly higher levels of intracellular glucose, TC, and TG compared to the blank group. Compared with the model group, the contents of glucose, TC, and TG in HepG2 cells were significantly reduced following the treatment with different concentrations of sanggenone D and kuwanon G, and no significant difference was shown between samples with different concentrations, which exhibited better inhibitory effect than the positive control basically.

### 3.8. Western Blot Analysis

**Figure 8:**
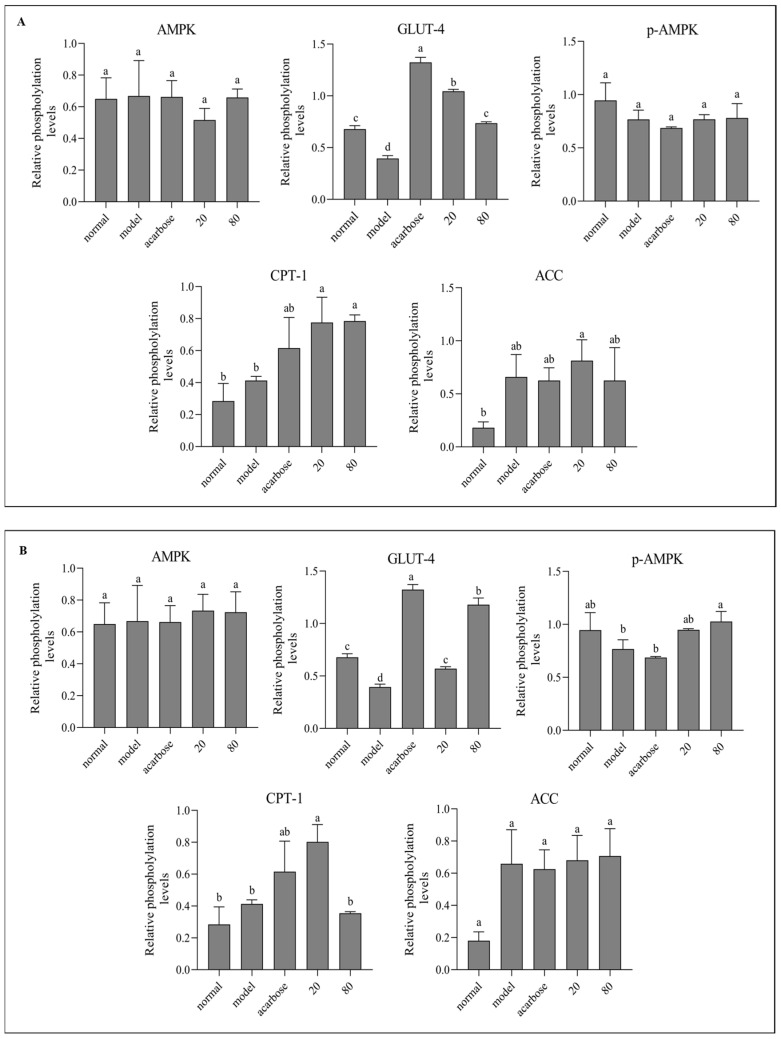

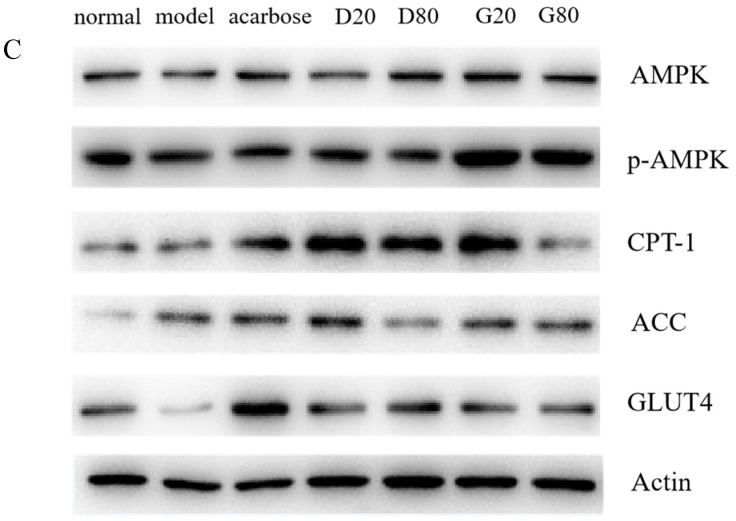
Effect of sanggenone D (**A**) and kuwanon G (**B**) on glucose transport-related mRNA expression in HepG2 cells, Western blot images of AMPK, p-AMPK, CPT-1, ACC, and GLUT4 (**C**). Note: Data are presented as mean ± standard deviation (SD) from n = 3 independent experiments. Different letters (a, b) indicate significant differences between groups (*p* < 0.05).

**Figure 9:**
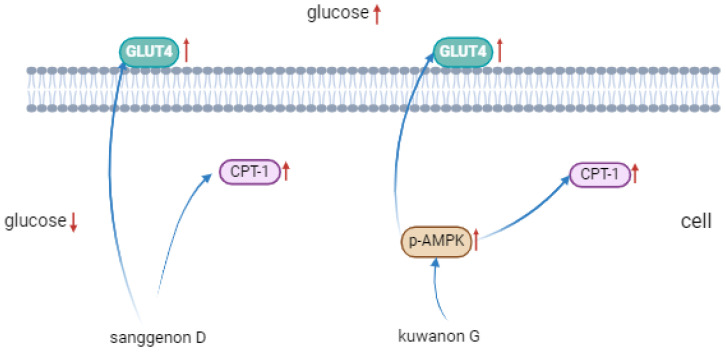
Mechanism of sanggenone D and kuwanon G effects on regulating glucose. Note: the upward arrow indicates that the content of this substance gradually increases with the addition of sanggenone D and kuwanon G.

The primary pathological basis of T2DM is insulin resistance, characterized by decreased insulin sensitivity and impaired glucose metabolism in insulin-targeted tissues [2]. Multiple pathways are involved in IR biological signal transduction. Adenosine 5′-monophosphate (AMP)-activated protein kinase (AMPK) is a metabolic regulator widely involved in the glycolipid metabolic process, and has become a new clinical treatment target for metabolic diseases based on IR [42]. Glucose transporter type 4 (GLUT4), an insulin-modulated glucose transporter in the AMPK pathway, enhances insulin sensitivity and glucose tolerance in adipose tissue when highly expressed [43]. Therefore, the corresponding dietary intervention targeting the AMPK–GLUT4 signaling pathway is beneficial for improving the diabetic metabolism and reducing insulin resistance. Studies have shown that polyphenols such as quercetin mainly enhance glucose absorption in muscle and adipose cells by activating the AMPK pathway, which translocates GLUT4 to the plasma membrane [44,45]. Korean red pepper polyphenol extract stimulated muscle cell uptake of glucose by activating AMPK, which regulated peroxisome proliferator-activated receptor gamma (PPAR-γ) and acetyl-CoA carboxylase (ACC) expression negatively [46]. p-Coumaric acid promoted the phosphorylation of AMPK, and increased the phosphorylation of ACC and the mRNA expression of carnitine palmitoyltransferase1 (CPT-1) in L6 skeletal muscle cells, thereby facilitating the β-oxidation of fatty acids, reducing the deposition of triglycerides, and promoting the glucose absorption of cells [47]. Figure 8 describes the protein expression levels of AMPK, GLUT4, p-AMPK, ACC, and CPT-1 in HepG2 cells after sample treatment with different concentrations. Compared with the model group, GLUT4 expression was notably up-regulated in both groups, and p-AMPK was significantly promoted in the kuwanon G group, indicating that AMPK phosphorylation was significantly activated in the kuwanon G group, which might be one of the reasons why kuwanon G obtained a better inhibitory effect than sanggenone D. In addition, both sanggenone D and kuwanon G significantly upregulated the expression of CPT-1, and CPT-1 could promote the decomposition and oxidation of fatty acids, which corresponded to the decrease in TC and TG results. The mechanism of action in regulating glucose metabolism is summarized in Figure 9.

## 4. Conclusions

The inhibition mechanisms and the structure–activity relationships of sanggenone D and kuwanon G on α-glucosidase were analyzed in this study. The findings revealed that both flavonoids effectively inhibited α-glucosidase activity, with kuwanon G exhibiting superior inhibitory potency compared to sanggenone D. This difference in potency was attributed to the cyclization of sanggenone D, which potentially weakened the activity of the isopentenyl group and eliminated a phenolic hydroxyl group, thereby reducing the formation of hydrogen bonds. Furthermore, fluorescence and FT-IR analyses indicated that both compounds changed the microenvironment and secondary structure of α-glucosidase through static quenching. Molecular docking results confirmed that the stability, binding energy, and affinity aligned with the inhibitory activity of the two compounds. The primary binding to α-glucosidase occurred spontaneously through hydrophobic interactions and hydrogen bonds with amino acid residues. In addition, the hypoglycemic potential of sanggenone D and kuwanon G was validated using a high-glucose-induced HepG2 cell model. Western blot analysis indicated that sanggenone D and kuwanon G regulated blood glucose, and the mechanism related to the activation of the GLUT4 pathway. These findings provide valuable insights into the potential of sanggenone D and kuwanon G as natural α-glucosidase inhibitors for diabetes management.

## Figures and Tables

**Table 1:** Km, Vm, Ki, and Kis values of the compounds inhibiting α-glucosidase activity.

	Concentration (μg/mL)	Km	Vm	Ki	Kis
Sanggenone D	0	78.49	5.02	252.95	9.86
	20	17.18	1.00		
	100	1.57	0.071		
	200	0.89	0.027		
Kuwanon G	0	1.73	0.27	83.46	50.89
	10	2.17	0.29		
	20	5.68	0.63		
	40	15.98	1.49		

**Table 2:** K_SV_, Kq, Ka, and n of compounds on α-glucosidase at three temperatures.

	T(K)	K_SV_ (×10^4^ L/mol)	Ra	Kq (×10^12^ L/mol)	Ka (×10^3^ L/mol)	n	Rb
Sanggenone D	298	3.90 ± 0.04	0.98	3.90 ± 0.04	7.28 ± 0.21	0.81	0.98
	304	3.24 ± 0.02	0.99	3.24 ±0.02	16.73 ± 0.14	0.93	0.99
	310	2.56 ± 0.02	0.98	2.56 ±0.02	180.18 ± 0.30	1.22	0.98
Kuwanon G	298	5.41 ± 0.01	0.99	5.41 ± 0.01	36.85 ± 0.17	0.96	0.99
	304	4.91 ± 0.03	0.99	4.91 ± 0.03	194.98 ± 0.42	1.15	0.99
	310	3.35 ± 0.01	0.98	3.35 ± 0.01	1984.27 ± 0.33	1.43	0.98

Note: Ra is the correlation coefficient for the K_SV_ value. Rb is the correlation coefficient for the Ka value.

**Table 3:** Binding free energies and energy components predicted by MM/GBSA (kcal/mol).

System Name	α-Glucosidase/Sanggenone D	α-Glucosidase/Kuwanon G
Δ*E_vdw_*	−22.55 ± 4.19	−26.16 ± 2.43
Δ*E*_elec_	109.95 ± 4.95	59.73 ± 5.51
ΔG_GB_	−94.59 ± 3.78	−44.70 ± 6.19
ΔG_SA_	−2.63 ± 0.51	−2.69 ± 0.26
ΔG_bind_	−9.83 ± 2.44	−13.83 ± 2.94

Δ*E_vdW_*: van der Waals energy. Δ*E_elec_*: electrostatic energy. Δ*G_GB_*: electrostatic contribution to solvation. Δ*G_SA_*: non-polar contribution to solvation. Δ*G_bind_*: binding free energy.

## Data Availability

The original contributions presented in this study are included in the article. Further inquiries can be directed to the corresponding author.

## Abbreviations

T2DM, type 2 diabetics; DMEM, Dulbecco’s modified Eagle’s medium; TC, total cholesterol; TG, triglyceride; TPEB, total protein extraction buffer; BCA, bicinchoninic acid assay; PVDF, polyvinylidene difluoride; PBST, phosphate buffered saline with Tween-20; RMSD, root mean square deviation; RMSF, root mean square fluctuation; AMPK, 5′-monophosphate AMP-activated protein kinase; GLUT4, glucose transporter type 4; PPAR-γ, peroxisome proliferator-activated receptor gamma; ACC, acetyl-CoA carboxylase; CPT-1, carnitine palmitoyltransferase1.
